# Minimal Change Disease in an Adult: A Case Report

**DOI:** 10.31729/jnma.7433

**Published:** 2022-04-30

**Authors:** Shashank Neupane, Prasamsa Pudasaini, Anupam Sharma, Shriya Sharma, Aakriti Adhikari, Kumar Roka

**Affiliations:** 1 Nepalese Army Institute of Health Sciences, Sanobharyang, Kathmandu, Nepal; 2 Department of Internal Medicine, Shree Birendra Hospital, Chhauni, Kathmandu, Nepal

**Keywords:** *electron microscopy*, *minimal change disease*, *nephrotic syndrome*, *prednisolone*

## Abstract

Minimal change disease is an important cause of nephrotic syndrome in children, however, few cases are seen among adults. There is very little literature regarding the occurrence of minimal change disease in adults. We reported a case of a 63-year-old male who presented with the complaint of swelling around the eyes mostly during the morning for 18 days and frothing of urine for 7 days. On examination, the patient was ill-looking and had edema around the eyes and over the ankles. After preliminary investigations, renal biopsy was performed and electron microscopy revealed diffuse effacement of foot processes of visceral epithelial cells suggesting minimal change disease (podocytopathy). The patient has been treated with tablet prednisolone 60 mg per oral once daily, tablet ramipril 2.5 mg per oral once daily, and tablet torsemide 20 mg per oral twice daily. Hence, minimal change disease should also be considered as a differential diagnosis in adults presenting with the features of nephrotic syndrome.

## INTRODUCTION

Minimal change disease (MCD) accounts for the vast majority of nephrotic syndrome in children, however only 15% of the cases in adults which is characterized by proteinuria, hypoalbuminemia, edema, and hypercholesterolemia.^1,2^ MCD can be primary (idiopathic) or secondary. The secondary causes such as medications like Non-steroidal Antiinflammatory Drugs (NSAIDs), hematologic or solid malignancies, infections, and other renal or systemic diseases account for up to 15% of minimal change disease in adults.^1^ Thus, it is crucial to obtain a detailed history of adults with MCD because the prognosis and management depend on the underlying etiology. Here, we present a case of a 63 years old male with MCD.

## CASE REPORT

A 63-year-old male presented to our hospital with a history of swelling around the eyes for 18 days which was insidious in onset, increased mostly during the morning, and later progressed to developing swelling over the ankles. He also complained of frothing of urine for 7 days which was insidious in onset and was also mostly seen in early morning urine aggravated by low intake of fluids. At the presentation, he was ill-looking, with edema over his eyes and ankle. There was no significant medical, family or psycho-social history. Soon a Reverse-Transcriptase Polymerase Chain Reaction (RT-PCR) was sent and turned out to be negative the next day. Similarly, initial investigations were sent to rule out causes of edema namely, renal, cardiac, endocrine, and nutritional causes which were unremarkable except for haemoglobin- 10.6 mg/dl, serum sodium- 116 meq/l, serum triglyceride- 201 mg/ dl, serum potassium- 2.9 meq/l and urinary albumin- +++. However, serum creatinine was within the normal range. The patient was then sent for renal biopsy. Ultrastructural examination showed:

1. Diffuse effacement of foot process of visceral epithelial cells.
2. Glomerular Basement Membrane (GBM) thickness varies from 280.9 to 506.3 nm (mean 308.9 nm)
3. There is no evidence of GBM structure abnormalities/reduplications.
4. Electron dense deposits are not identified in GBM or mesangial areas.

The biopsy shows non-proliferative glomerular morphology, Direct Immunofluorescence (DIF) studies do not reveal significant glomerular immune staining, and electron microscopy studies have shown diffuse effacement of visceral epithelial cells foot processes, suggesting a primary podocytopathy/diffuse podocyte injury (Minimal Change Disease) in the present clinical context.

The patient was treated with tablet Prednisolone 60 mg per oral once daily, tablet Ramipril 2.5 mg per oral once daily, and tablet Torsemide 20 mg per oral twice daily. Despite our full effort and unresponsiveness of patient we could not acquire the outcome of trial of prednisolone in our patient. We have no information in regard to remission or steroid dependence or steroid resistance our patient had developed following treatment with steroid.

## DISCUSSION

Minimal change disease is idiopathic but can have secondary causes such as exposure to other agents like infections (i.e. tuberculosis, syphilis, mycoplasma, ehrlichiosis, hepatitis C virus), neoplasms (i.e. haematological malignancies such as leukaemia), Hodgkin and non-Hodgkin lymphoma); allergy (i.e. bee and medusa stings, cat fur, fungi, poison ivy, ragweed pollen, house dust), drugs (i.e. NSAIDs, lithium, antibiotics, immunizations, and gamma interferon), and other glomerular diseases associated with IgA nephropathy, Systemic Lupus Erythematosus (SLE), type 1 Diabetes Mellitus (DM) and Human Immunodeficiency Virus (HIV).^3^ The patient in our case was taking Naproxen 500 mg twice a day for rheumatoid arthritis. Similarly, a CT abdomen was carried out which turned out to be insignificant. Serology for hepatitis B, Hepatitis C, and HIV was non-reactive. Based on the history of long-term use of NSAIDS for rheumatoid arthritis, the secondary for minimal change disease induced by NSAIDs can be established.

The clinical presentation of minimal change disease includes oedema, nephrotic-range proteinuria, hypoalbuminemia, and hyperlipidemia.^4^ Patient in this case report also presented with oedema around the eyes and over the ankles. On urinalysis, 10%-30% of adults with MCD presented with microscopic hematuria which may resolve with disease remission.^4^ However, the patient did not have microscopic hematuria but frothing of urine.

The pharmacologic treatment for MCD includes corticosteroids such as prednisone or prednisolone which induce immunosuppression and decrease the activity of T-cell cytokines.^5^ MCD in children usually remits within a few weeks of starting corticosteroids, however, adult MCD responds less rapidly. Three to four months of steroid therapy may be required to induce remission. On the other hand, 10%-30% of adults may fail to respond to steroid therapy.^4^ Such cases can be treated with immunosuppressive medications such as cyclosporine.^5^ The patient was treated with tablet prednisolone 60 mg per oral once daily, tablet ramipril 2.5 mg per oral once daily, and tablet torsemide 20 mg per oral twice daily.

It is equally important to educate the patient about kidney disease in order to increase compliance to the treatment, to educate about the dietary plan i.e. to follow a diet low in sodium, to be aware of their calorie intake to prevent further weight gain as steroid can cause weight gain and to be careful about taking over-the-counter medications such as NSAIDs because they can be nephrotoxic.^5^

In adult-onset minimal change disease, the longterm prognosis is excellent, as 75%-90% of treated patients remain in remission however, progression of the disease to end-stage renal disease occurs in approximately less than 5% of the adults and is often due to an alternative diagnosis of Focal Segmental Glomerulosclerosis (FSGS).^6^

## References

[1] Nicholas PD, Garrahy I (2019). Adult minimal change disease with acute kidney injury: a case report and literature review.. J Community Hosp Intern Med Perspect..

[2] Saleem MA, Kobayashi Y (2016). Cell biology and genetics of minimal change disease.. F1000Res..

[3] Zamora G, Pearson-Shaver AL (2021). Minimal Change Disease [Internet]..

[4] Hogan J, Radhakrishnan J (2013). The treatment of minimal change disease in adults.. J Am Soc Nephrol..

[5] Mitchell-Brown FM, Veisze T (2019). Minimal change disease: A case report.. Nursing..

[6] Korbet SM, Whittier WL (2019). Management of Adult Minimal Change Disease.. Clin J Am Soc Nephrol..

